# Bioinformatics-based identification and validation of hub genes associated with aging in patients with coronary artery disease

**DOI:** 10.18632/aging.205309

**Published:** 2023-12-14

**Authors:** Wangmeng Zhang, Minmin Zhao, Li Xin, Ximei Qi, Ping Cao, Jiyan Wang, Xin Li

**Affiliations:** 1 Department of Obstetrics, The Affiliated Tai’an City Central Hospital of Qingdao University, Tai’an 271000, Shandong, China; 2 Department of Cardiology, The Affiliated Tai’an City Central Hospital of Qingdao University, Tai’an 271000, Shandong, China; 3 Department of Geriatrics, The Affiliated Tai’an City Central Hospital of Qingdao University, Tai’an 271000, Shandong, China; 4 Department of Internal Medicine, The Fourth People's Hospital of Tai’an City, Tai’an 271000, Shandong, China; 5 Department of Obstetrics, Tai’an Maternal and Child Health Care Hospital, Tai’an 271000, Shandong, China

**Keywords:** coronary artery disease, aging, bioinformatics, *HSP90AA1*, *CEBPA*

## Abstract

Coronary artery disease (CAD) is the most common aging-related disease in adults. We used bioinformatics analysis to study genes associated with aging in patients with CAD. The microarray data of the GSE12288 dataset were downloaded from the Gene Expression Omnibus database to obtain 934 CAD-associated differentially expressed genes. By overlaying them with aging-related genes in the Aging Atlas database, 33 differentially expressed aging-related genes (DEARGs) were identified. Gene Ontology and Kyoto Encyclopedia of Genes and Genomes enrichment analyses revealed that the 33 DEARGs were mainly enriched in cell adhesion and activation, Th17 and Th1/Th2 cell differentiation, and longevity regulation pathways. Hub genes were further screened using multiple algorithms of Cytoscape software and validation set GSE71226. Clinical samples were then collected, and the expression of hub genes in whole blood was detected by real-time quantitative polymerase chain reaction, enzyme-linked immunosorbent assay, and western blot at the transcription and translation levels. Finally, *HSP90AA1* and *CEBPA* were identified as hub genes. The results of this study suggest that *HSP90AA1* and *CEBPA* are closely related to CAD. These findings provide a theoretical basis for the association between aging effectors and CAD, and indicate that these genes may be promising biomarkers for the diagnosis and treatment of CAD.

## INTRODUCTION

Coronary artery disease (CAD), also known as coronary heart disease, is the most common aging-related disease in adults [1, 2]. Despite advances in treatment and lifestyle modifications, CAD and its complications remain the most common causes of morbidity and mortality worldwide [3]. Advanced age; smoking; hypertension; high levels of low-density lipoprotein, cholesterol and fat; and diabetes all contribute to CAD [4, 5], adversely affecting human life expectancy.

Aging is the basis of organ function decline and the main risk factor for numerous diseases, including neurodegenerative diseases, cardiovascular and cerebrovascular diseases, and tumors [6, 7]. China’s aging population is growing; the number of senior citizens is expected to reach 480 million by 2050, accounting for approximately 25% of Asia’s elderly population by that time [8]. The incidence of several age-related diseases, including cardiovascular diseases [9], is increasing on an annual basis. This trend is making life more challenging for older individuals and placing a significant economic burden on society. Thus, there is an urgent need to perform research on the aging process, explore the molecular mechanism of the occurrence and development of aging-related diseases, and identify therapeutic targets for aging-related diseases. The correlation between CAD and aging-related genes (ARGs) is unclear, and which ARGs are essential for the development of CAD is unknown. Further research is needed to identify therapeutic biomarkers for CAD based on potential ARGs.

**Figure 1:**
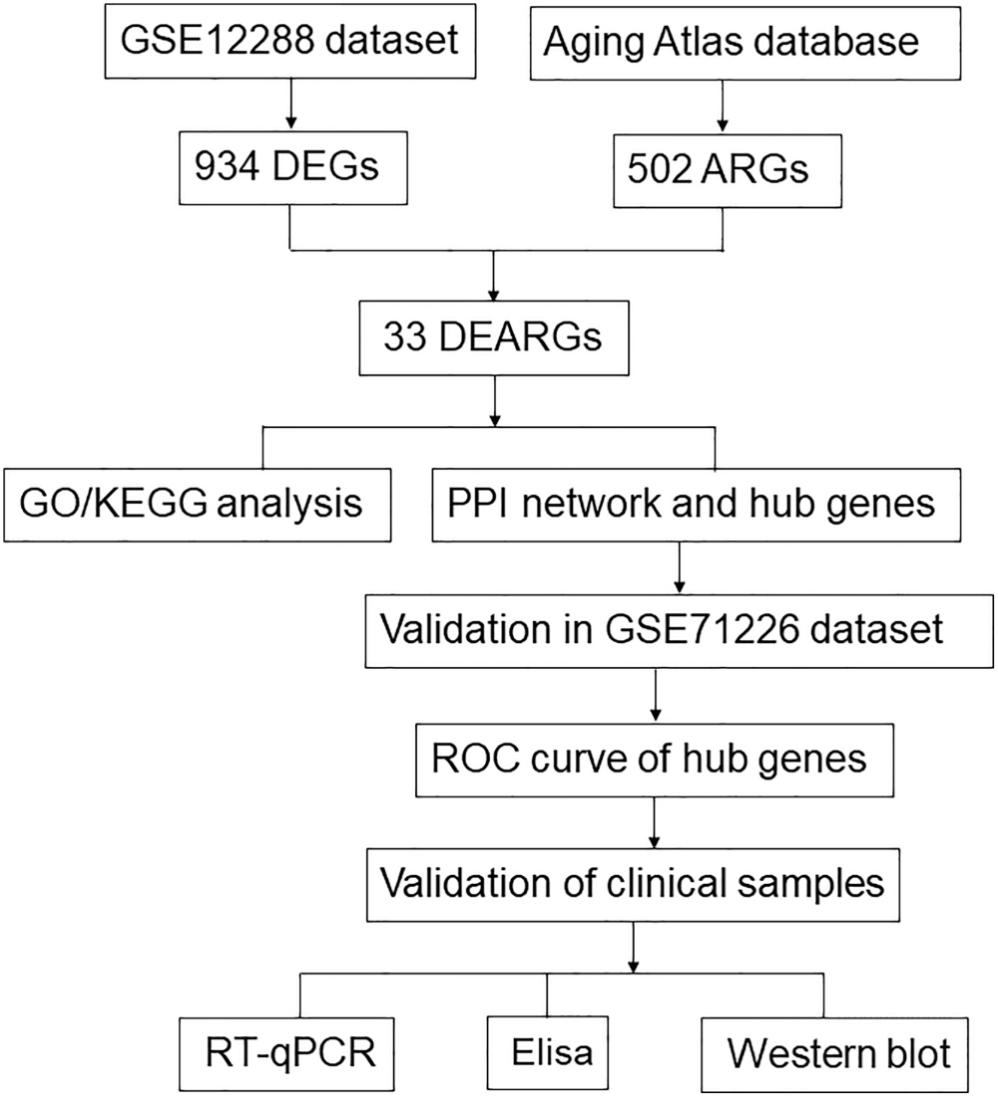
Study flow chart.

In this study, we examined the relationship between ARGs and CAD using bioinformatics methods for the first time. We screened differentially expressed ARGs (DEARGs) based on the Gene Expression Omnibus (GEO) and Aging Atlas database. Gene Ontology (GO) enrichment analysis, Kyoto Encyclopedia of Genes and Genomes (KEGG) enrichment analysis, and protein–protein interaction (PPI) analysis were performed on the DEARGs. In addition, we used multiple algorithms from Cytoscape software to screen hub genes and validate them with a separate dataset. Blood samples were collected, and the mRNA and protein expression levels of hub genes in whole blood from patients in the CAD group and control group were detected by real-time quantitative polymerase chain reaction (RT-qPCR), enzyme-linked immunosorbent assay (ELISA), and western blot. Finally, the hub genes of DEARGs in CAD were identified. A flow chart of this study is shown in Figure 1.

## RESULTS

### Screening of candidate DEARGs in CAD

**Figure 2:**
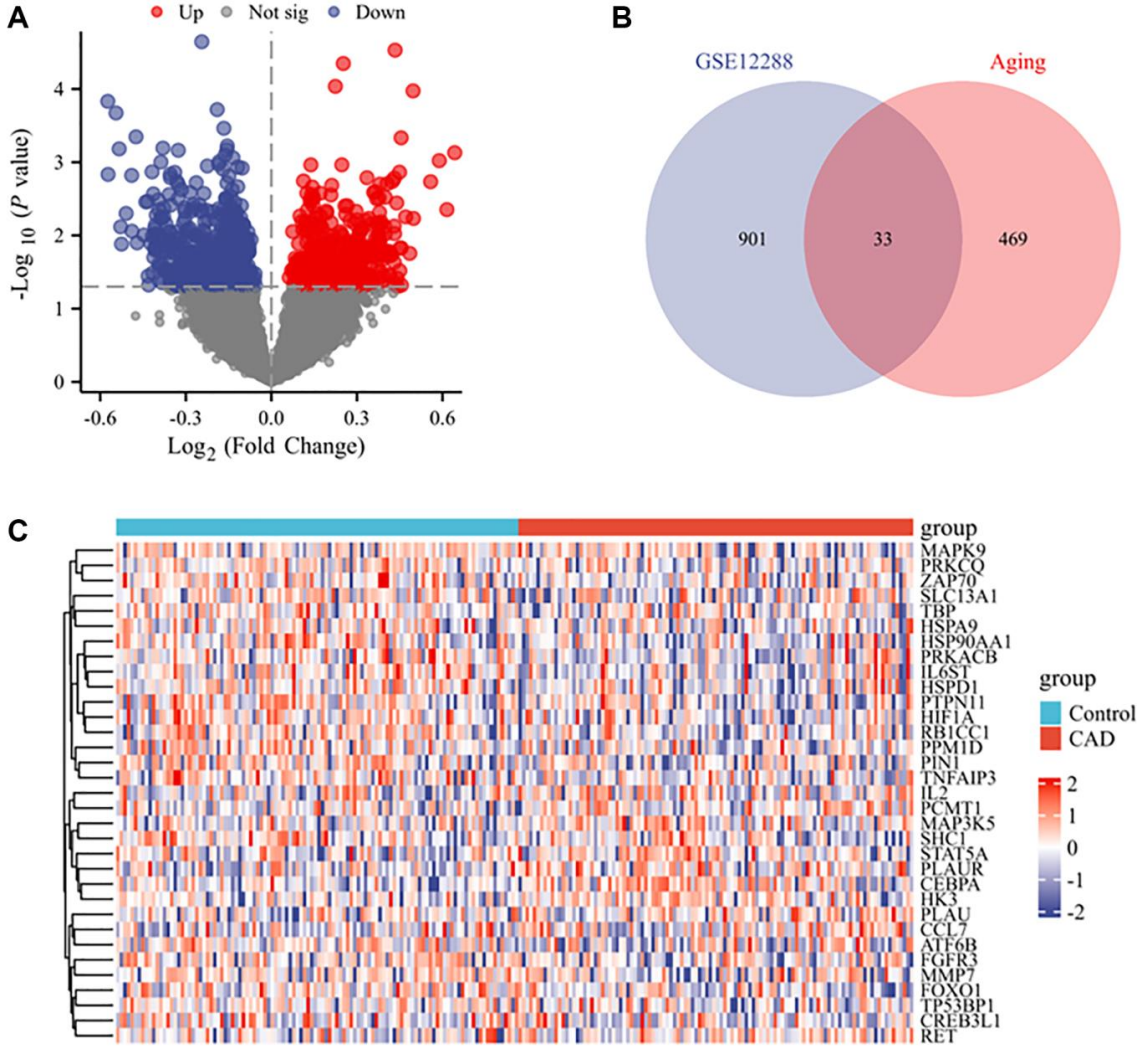
**DEARGs between CAD and control groups.** (**A**) Volcano plot of the DEGs from GSE12288 with *P* < 0.05 as the threshold value. Red and blue dots indicate significantly upregulated and downregulated genes, respectively. (**B**) Venn diagram of CAD DEGs and ARGs. (**C**) Heatmap of the expression of 33 ARGs and CAD-related genes.

In the GSE12288 dataset, 934 differentially expressed genes (DEGs) (404 upregulated and 530 downregulated) were identified between CAD and control samples, with *P* < 0.05 as the cut-off value (Figure 2A). These 934 DEGs were compared with 502 ARGs in the Aging Atlas database, and 33 DEARGs were identified in both datasets (Figure 2B). Among the 33 genes, 10 genes were upregulated (*IL2*, *CCL7*, *PLAU*, *CEBPA*, *RET*, *MAP3K5*, *HK3*, *PLAUR*, *PCMT1*, and *STAT5A*) and 23 genes were downregulated (*SLC13A1*, *PIN1*, *FGFR3*, *MMP7*, *ATF6B*, *MAPK9*, *CREB3L1*, *IL6ST*, *HIF1A*, *PRKACB*, *PTPN11*, *TBP*, *RB1CC1*, *PRKCQ*, *FOXO1*, *HSP90AA1*, *ZAP70*, *PPM1D*, *TP53BP1*, *TNFAIP3*, *HSPA9*, *HSPD1*, and *SHC1*) (Figure 2C).

### Functional enrichment analysis of DEARGs

**Figure 3:**
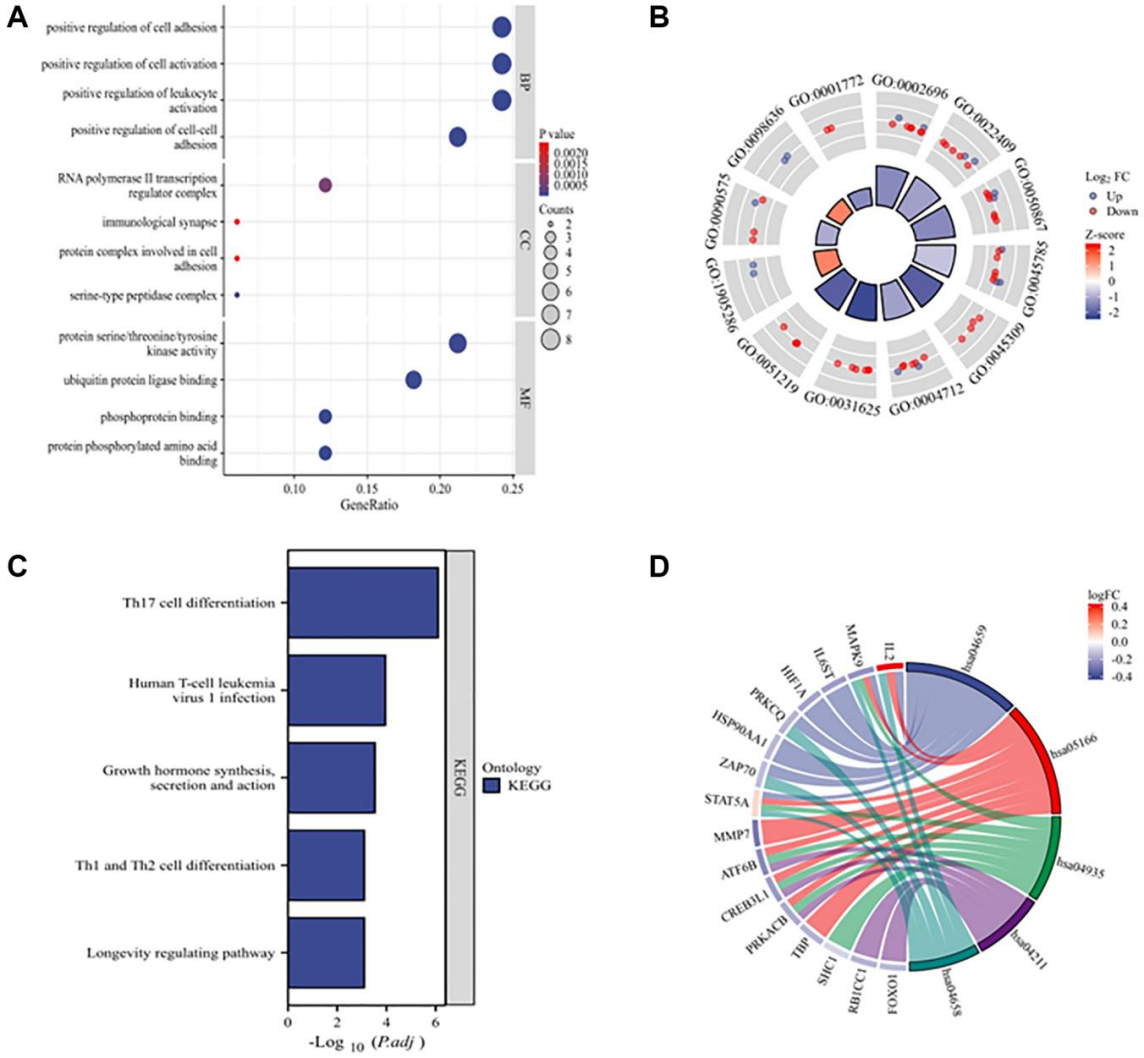
**GO and KEGG enrichment analyses of 33 DEARGs.** (**A**) Bubble plot of enriched GO terms. (**B**) The circle plot shows the top 12 GO terms. The inner circle represents the z-scores, and the outer circle represents the number of genes in the GO terms. Red indicates upregulated ARGs, and green indicates downregulated ARGs. (**C**) KEGG pathways of DEARGs. (**D**) Chord plot of enriched KEGG pathways. Abbreviations: BP: biological process; CC: cellular component; MF: molecular function.

The functional enrichment analysis chart of the DEARGs shows the four most significantly enriched GO terms (Figure 3A, 3B). The enriched biological processes include positive regulation of cell adhesion, positive regulation of cell activation, positive regulation of leukocyte activation, and positive regulation of cell–cell adhesion. The enriched cellular components include RNA polymerase II transcription regulator complex, immunological synapses, protein complexes involved in cell adhesion, and serine-type peptidase complex. The enriched molecular functions are protein serine/threonine/tyrosine kinase activity, ubiquitin protein ligase binding, phosphoprotein binding, and protein phosphorylated amino acid binding. The KEGG enrichment analysis revealed that DEARGs play key roles in Th17 cell differentiation; human T-cell leukemia virus 1 infection; growth hormone synthesis, secretion, and action; Th1 and Th2 cell differentiation; and longevity-regulating pathways (Figure 3C, 3D).

### Analysis of PPI networks and identification of hub genes

**Figure 4:**
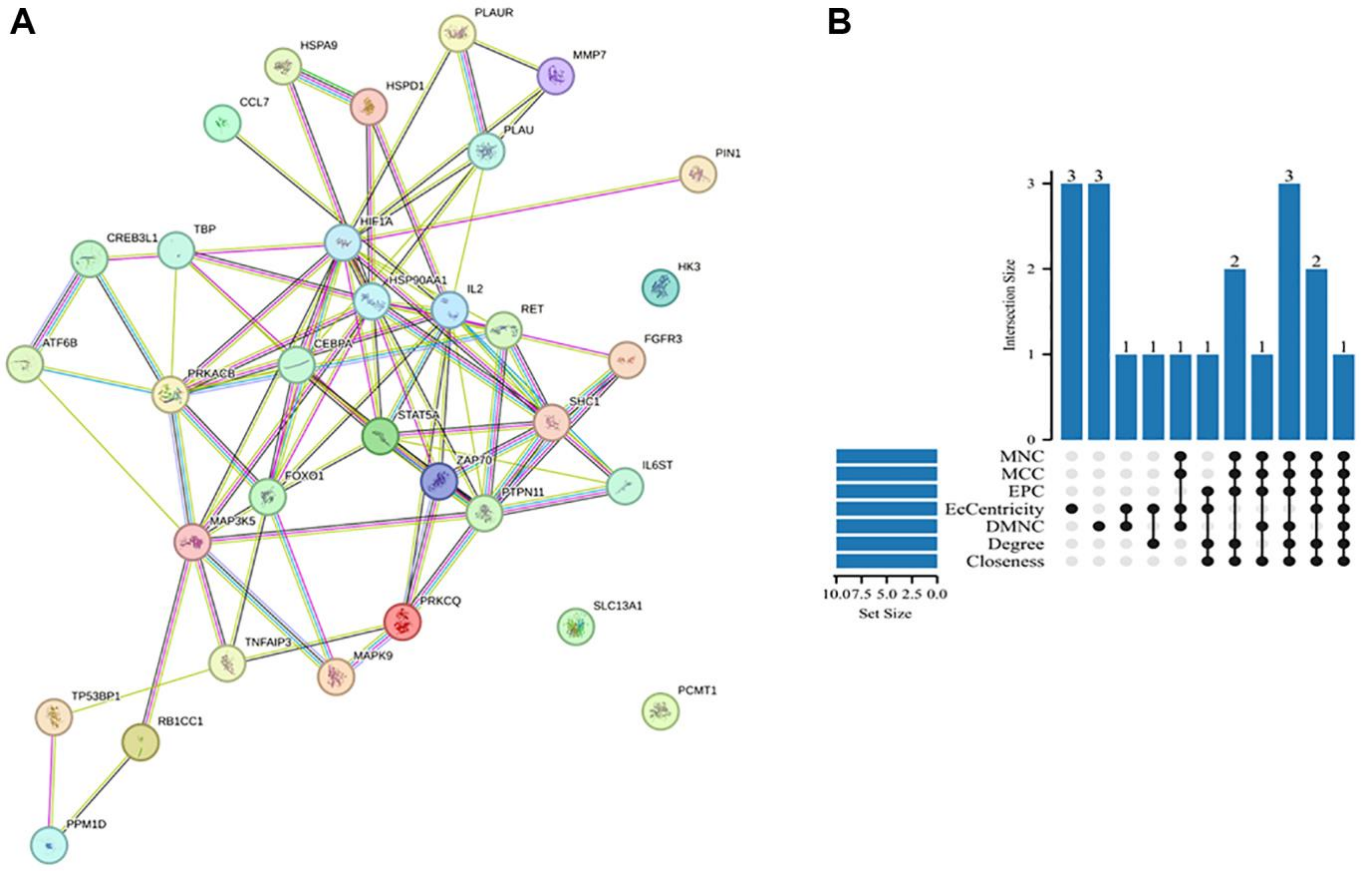
**PPI network and hub genes.** (**A**) PPI network analysis of DEARGs in the GSE12288 dataset. The circles represent genes, the lines represent PPIs between genes, and the results in the circles represent the protein structures. The colors of the lines represent evidence of PPIs. (**B**) UpSet map obtained by crossing the hub genes generated by the seven algorithms.

PPI networks were constructed using the STRING database to identify interactions between DEARGs and further visualize the results comprising 33 nodes and 86 edges. Figure 4A shows the PPI network of the DEARGs. The circles represent genes, the lines represent PPIs between genes, and the results in the circles represent the protein structure. The colors of the lines represent evidence of PPIs. We used various algorithms from the Cytoscape software Cytohubba plugin, including Degree, MCC, MNC, EPC, DMNC, EcCentricity, and Closeness, to identify hub genes. We then created an UpSet map to visualize the results (Figure 4B). Finally, six hub genes were obtained: *CEBPA*, *HIF1A*, *HSP90AA1*, *IL2*, *FOXO1*, and *STAT5A* (Table 1).

**Table 1:** Hub genes were screened using 7 algorithms of Cytoscape software Cytohubba plugin.

**Elements**	**Closeness**	**Degree**	**DMNC**	**EcCentricity**	**EPC**	**MCC**	**MNC**
***CEBPA***	1	1	1	1	1	1	1
***HSP90AA1***	1	1	0	1	1	1	1
***IL2***	1	1	1	0	1	1	1
***FOXO1***	1	1	1	0	1	1	1
***HIF1A***	1	1	0	1	1	1	1
***STAT5A***	1	1	1	0	1	1	1
*MAP3K5*	1	1	0	1	1	0	0
*ZAP70*	1	0	1	0	1	1	1
*PRKACB*	0	1	0	1	0	0	0
*IL6ST*	0	0	1	1	0	1	1
*PLAU*	0	0	1	1	0	0	0
*PLAUR*	0	0	1	0	0	0	0
*FGFR3*	0	0	1	0	0	0	0
*MMP7*	0	0	1	0	0	0	0
*MAPK9*	0	0	0	1	0	0	0
*CREB3L1*	0	0	0	1	0	0	0
*RET*	0	0	0	1	0	0	0
*SHC1*	1	1	0	0	1	1	1
*PTPN11*	1	1	0	0	1	1	1

### Validation of differentially expressed hub genes in GSE datasets

**Figure 5:**
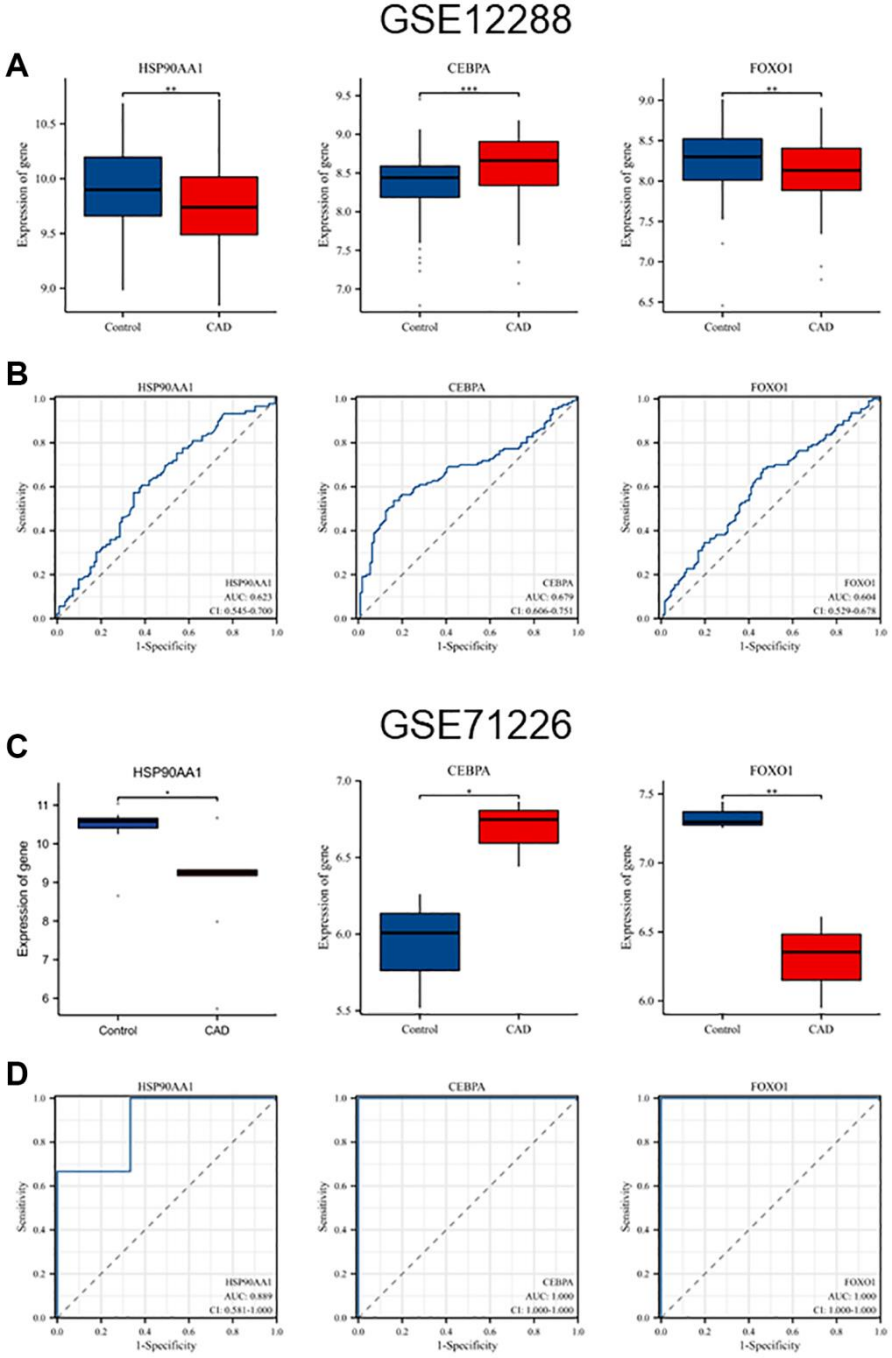
**Validation of hub genes expression levels and diagnostic value in GSE12288 and GSE71226 datasets.** (**A**) Differential expression of *HSP90AA1*, *CEBPA*, and *FOXO1* in GSE12288. (**B**) ROC curves of *HSP90AA1*, *CEBPA*, and *FOXO1* in GSE12288. (**C**) Differential expression of *HSP90AA1*, *CEBPA*, and *FOXO1* in GSE71226. (**D**) ROC curves of *HSP90AA1*, *CEBPA*, and *FOXO1* in GSE71226.

The differences in the expression of the six hub genes in GSE12288 and GSE71226 are shown in Table 2. Among them, *HSP90AA1*, *CEBPA*, and *FOXO1* were differentially expressed in the GSE71226 dataset, consistent with GSE12288, whereas *HIF1A*, *IL2*, and *STAT5A* were not differentially expressed. Figure 5A, 5C shows the differences in the expression levels of *HSP90AA1*, *CEBPA*, and *FOXO1* between the CAD and control groups in the GSE12288 and GES71226 datasets. Receiver operating characteristic (ROC) curves were used to detect the diagnostic value of three hub genes for CAD in two datasets (Figure 5B, 5D). *HSP90AA1* (area under the curve (AUC), 0.889), *CEBPA* (AUC, 1.000), and *FOXO1* (AUC, 1.000) were detected in GSE71226 with high accuracy.

**Table 2:** The analysis of 6 aging- and CAD-related genes in GSE12288 and GSE71226 datasets.

**Gene**	**GSE12288**		**GSE71226**	
	***P* value**	**Type**	***P* value**	**Type**
***CEBPA***	0.000	Up	0.039	Up
***FOXO1***	0.010	Down	0.007	Down
***HSP90AA1***	0.007	Down	0.046	Down
*HIF1A*	0.001	Down	0.259	—
*IL2*	0.023	Up	0.240	—
*STAT5A*	0.029	Up	0.100	—

### Validation of hub genes at the transcriptional level

**Figure 6:**
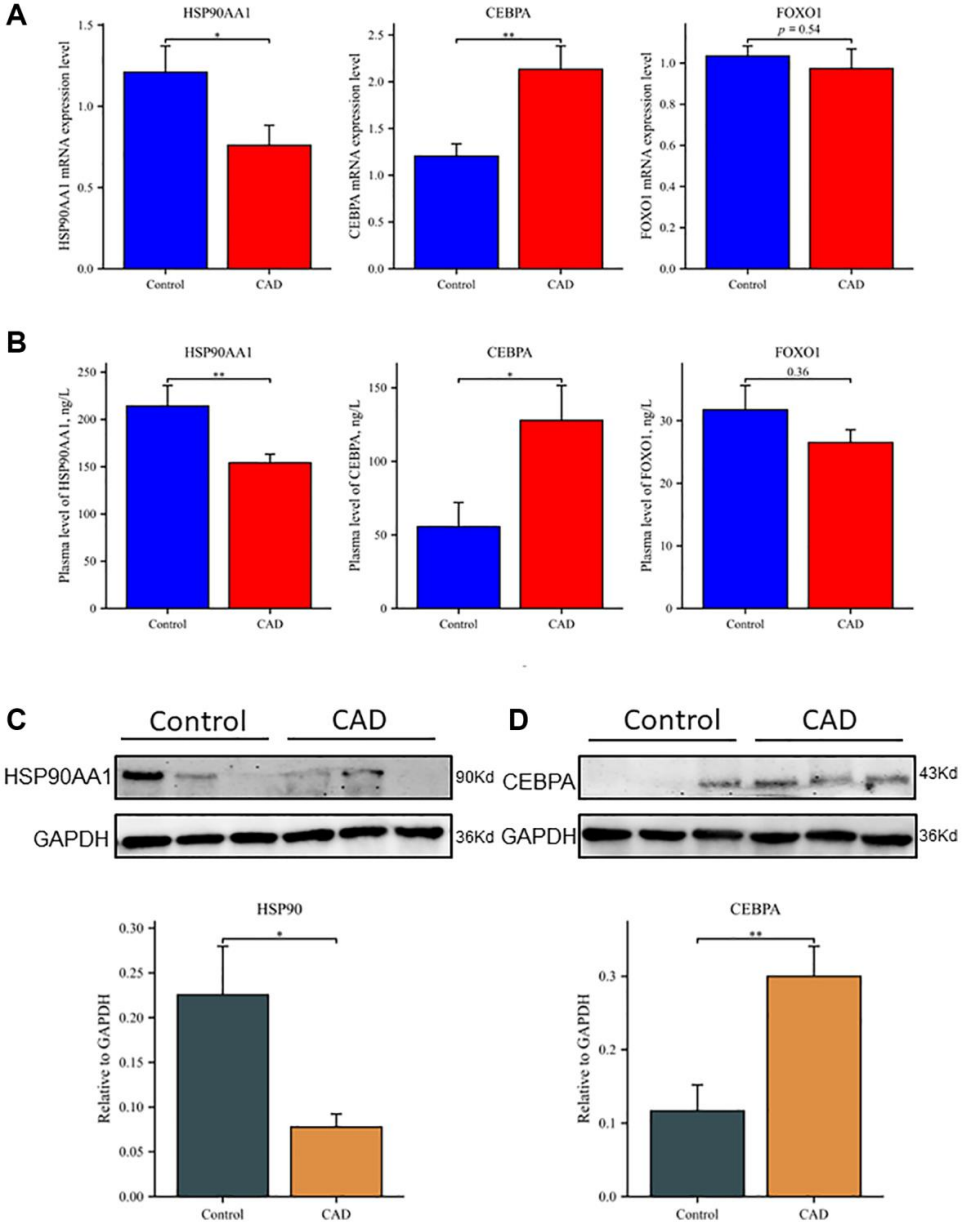
**Differential expression of hub genes at transcription and translation levels in clinical samples.** (**A**) Relative mRNA levels of *HSP90AA1*, *CEBPA*, and *FOXO1* by RT-qPCR analysis in whole blood among controls (*n* = 50) and patients with CAD (*n* = 50). (**B**) Plasma expression levels of *HSP90AA1*, *CEBPA*, and *FOXO1* in controls and patients with CAD. (**C**) Western blot analyses of *HSP90AA1* protein levels in PBMCs, including representative blot images and a densitometric summary of the blot analysis after normalization to GAPDH. (**D**) Western blot analyses of *CEBPA* protein levels in PBMCs, including representative blot images and a densitometric summary of the blot analysis after normalization to GAPDH. ^*^P < 0.05, ^**^P < 0.01. Vertical bars represent standard error.

The mRNA expression levels of *HSP90AA1*, *CEBPA*, and *FOXO1* in whole blood of the CAD group and control group were detected in 100 clinical samples. Figure 6A shows that the mRNA expression level of *HSP90AA1* was lower (*P* < 0.05) and that the mRNA expression level of *CEBPA* (*P* < 0.01) was higher in the CAD group than in the control group. These results are consistent with the GSE12288 and GSE71226 datasets. There was no significant difference in *FOXO1* expression between the two groups (*P* = 0.54).

### Validation of hub genes at the translational level

The ELISA results showed that the plasma levels of *HSP90AA1*, *CEBPA*, and *FOXO1* were consistent with mRNA expression (Figure 6B). The levels of *HSP90AA1* in plasma samples of the control group and CAD group were 214.18 ± 135.33 and 154.08 ± 57.13 ng/L, respectively (*P* < 0.01). The plasma *CEBPA* concentration was significantly higher in the CAD group than in the control group (127.78 ± 134.37 vs. 55.56 ± 85.59 ng/L, respectively; *P* < 0.05). The expression level of *FOXO1* was lower in the CAD group than in the control group (26.51 ± 14.46 vs. 31.74 ± 27.42 ng/L, respectively), but the difference was not statistically significant (*P* = 0.36). Western blot analysis showed that the level of *HSP90AA1* protein in peripheral blood mononuclear cells (PBMCs) was lower in the CAD group than in the control group (Figure 6C), while the level of *CEBPA* protein was significantly higher in the CAD group than in the control group (Figure 6D). Possibly due to the low expression of *FOXO1* in PBMCs, we were unable to visualize *FOXO1* with western blot and therefore could not evaluate it.

### *HSP90AA1* and *CEBPA* were involved in the most significantly enriched GO and KEGG pathways

In the most enriched GO terms, *CEBPA* participated in positive regulation of leukocyte activation, positive regulation of cell activation, and RNA polymerase II transcription regulator complex, whereas *HSP90AA1* participated in ubiquitin protein ligase binding. *HSP90AA1* was also involved in the most enriched KEGG pathway, namely Th17 cell differentiation (Table 3).

**Table 3:** The most significantly enriched GO terms and KEGG pathway analysis involved in *HSP90AA1* and *CEBPA*.

**ID**	**Description**	***P* value**	**Hub gene**
GO:0002696	Positive regulation of leukocyte activation	5.0285E-07	*CEBPA*
GO:0050867	Positive regulation of cell activation	6.5504E-07	*CEBPA*
GO:0090575	RNA polymerase II transcription regulator complex	0.0005793	*CEBPA*
GO:0031625	Ubiquitin protein ligase binding	1.3112E-05	*HSP90AA1*
hsa04659	Th17 cell differentiation	4.4253E-09	*HSP90AA1*

## DISCUSSION

With the gradual aging of the worldwide population, it is becoming more critical to study and formulate healthy aging strategies. Aging is a multifactorial progressive process influenced by genetic and epigenetic regulation, post-translational regulation, host–microbiome interactions, metabolic regulation, lifestyle, and many other factors [10–14]. The Aging Atlas database used in this study focuses on big data generated by omics techniques, providing a wider range of valuable resources for the aging research community and other life scientists [15]. The major risk factor for cardiovascular disease is aging [16]. The study of ARGs associated with CAD has important clinical significance and social value.

In this study, DEARGs were identified for the first time by bioinformatics analysis, and the role of ARGs in the pathogenesis of CAD was discussed in the context of such analysis. We screened 33 DEARGs of CAD from the GEO dataset and Aging Atlas database. The GO analysis revealed that DEARGs were enriched in processes related to positive regulation of cell adhesion and protein serine/threonine/tyrosine kinase activity. KEGG pathway analysis showed that DEARGs were involved in Th17 cell differentiation; human T-cell leukemia virus 1 infection; growth hormone synthesis, secretion, and action; Th1 and Th2 cell differentiation; and longevity-regulating pathways.

Six hub genes (*HSP90AA1*, *IL2*, *CEBPA*, *FOXO1*, *HIF1A*, and *STAT5A*) were obtained using seven algorithms in the Cytoscape plugin. *HSP90AA1*, *CEBPA*, and *FOXO1*, which exhibited consistently differential expression, were selected based on analysis of a single GEO dataset. Clinical sample verification revealed no statistically significant difference in the expression of *FOXO1* between the CAD and control groups, and the gene could not be detected by western blot because of low expression in PBMCs. Therefore, *HSP90AA1* and *CEBPA* were identified as the final hub genes. *HSP90AA1* is associated with the most significantly enriched GO term (ubiquitin protein ligase binding) and the most enriched KEGG pathway (Th17 cell differentiation). The significantly enriched GO items in which *CEBPA* participates are positive regulation of leukocyte activation, positive regulation of cell activation, and RNA polymerase II transcription regulator complex.

Heat shock proteins (HSPs) are a class of highly conserved stress proteins that have molecular chaperone activity and are involved in various aspects of protein biogenesis, including folding, oligomer assembly, transport to specific subcellular compartments, and controlled switching between active/inactive conformations [17]. Furthermore, HSPs are tightly controlled by cellular regulatory mechanisms that protect cells and tissues from the misfolding of denatured proteins by regulating transcription and translation [18]. HSP90AA1 is the most widely studied member of the HSP family, and its primary role is to maintain protein homeostasis and protect cells. Animal experiments have confirmed that HSP90 (AA1) plays an important role in myocardial ischemia/reperfusion (I/R) injury and cardiac protection [19–23]. HSP90 (AA1)-mediated anti-apoptosis plays a crucial role in preconditioning cardiac protection [20, 21]. Liraglutide preconditioning elevates HSP90AA1 levels, inhibits the inflammatory response and C5a/NF-κB signaling, alleviates I/R-induced cardiocyte apoptosis, and protects the heart [24]. According to a study by Zhu et al. [25], knockdown of HSP90AA1 can enhance the cardiomyocyte apoptosis induced by oxygen–glucose deprivation, and inhibition of miR-1 can lead to increases in HSP90AA1 and Bcl-2; these processes are conducive to protection against myocardial I/R injury. HSP90AA1 also plays a relevant role in longevity. HSP90AA1-mediated regulation of a mammalian transcription factor EB ortholog was found to be involved in the extended lifespan of *Caenorhabditis elegans* in the absence of its food source bacteria [26].

CEBPA is a myeloid transcription factor, and mutations in this protein play a crucial role in the pathogenesis of hematologic tumors [27, 28]. However, such mutations have not been thoroughly studied in cardiovascular disease and in promoting atherosclerosis. One study showed that CEBPA mediates epicardial activation during heart development and injury and that disruption of CEBPA signaling in the epicardial tissue in adults reduces injury-induced neutrophil infiltration and improves cardiac function [29]. Together with PPARγ, CEBPA regulates the adipogenesis process and is involved in the sequence expression of adipocyte-specific proteins [30–36]. CEBPA is upregulated in unstable plaques, and overexpression of CEBPA may contribute to the occurrence and development of atherosclerosis [37, 38]. However, Bristol et al. [39] observed that CEBPA and CEBPB bind the TNFR1 promoter, increase its expression through a positive feedback mechanism, and induce an increase in TNF expression. TNF-α promotes the development of atherosclerosis [40–42]. CEBPA has also been studied in relation to aging. Aging exacerbates acute and chronic alcohol-induced liver injury in mice and humans by inhibiting the sirtuin 1-CEBPA-mirNA-223 axis of neutrophils [43]. Podocyte CEBPA deficiency can aggravate podocyte senescence and kidney injury in senescent mice [44].

In summary, 33 DEARGs were identified between the control and CAD samples by bioinformatics analysis. Based on the dataset and clinical sample validation, *HSP90AA1* and *CEBPA* were the final hub genes. The results of this study suggest that *HSP90AA1* and *CEBPA* are closely related to CAD, providing a theoretical basis for the association between aging effectors and CAD. These genes may be promising biomarkers for the diagnosis and treatment of CAD.

## MATERIALS AND METHODS

### Aging-related database and microarray data

In total, 502 ARGs were obtained from the Aging Atlas database (https://ngdc.cncb.ac.cn/). The microarray was downloaded from the NCBI GEO database containing two transcription profiles (GSE12288 and GSE71226.) The GSE12288 dataset was used as a training set and included 110 patients with CAD and 112 healthy individuals. The GSE71226 dataset was used as the validation set.

### Analysis of DEARGs

The DEGs from the CAD group and the control group within the GSE12288 dataset were analyzed using the R language software package limma (version 4.2.1). Genes in each sample that met the criteria of *P* < 0.05 were retained. The results of the differential expression analysis were visualized using the “ggplot2” package to create volcano plots. The DEGs were then compared with 502 ARGs to identify DEARGs. The specific and overlapping components of DEGs and ARGs were analyzed, and the results were visualized as Venn diagrams using the “ggplot2” and “VennDiagram” packages. Furthermore, the “ComplexHeatmap” package was used to create a heatmap representing the expression patterns of DEARGs.

### GO and KEGG

GO functional annotation refers to the use of standard expression terms to describe the biological function of genes and proteins in different databases. GO annotations contain three aspects of biological content: Biological Processes, Cellular Components, and Molecular Functions [45]. KEGG is a bioinformatics resource on genomes that establishes links from the collection of genes within the genome to the high-level functioning of cells and organisms [46]. GO and KEGG enrichment analyses were performed using R software to determine the potential biological function of these DEARGs in CAD. Specifically, the R software “ClusterProfiler” package was used for enrichment analysis after ID conversion of the input DEARGs list, and the “ggplot2” package was used for visualization of the enrichment analysis results.

### PPI network and hub genes

The list of proteins was uploaded to an online website called STRING (https://string-db.org/), thereby building the PPI network to analyze the internal connections among DEARGs. Using the Cytohubba plugin in Cytoscape software, 7 algorithms (Closeness, Degree, DMNC, EcCentricity, EPC, MCC, and MNC) were selected to obtain the top 10 genes in each algorithm. The unique and common parts of each group of data were analyzed. The “ggplot” package was used to create an UpSet diagram for visualization of the results. We screened the first six genes as hub genes for further research.

### Validation of hub genes and mapping of ROC curve

We used the validation set GSE71226 to verify whether the differential expression of hub genes was consistent with GSE12288, and we screened out genes with the same differential expression. Differences in the expression levels of hub genes between the control group and the CAD group were further verified in the GSE12288 and GSE71226 datasets, and the diagnostic value of hub genes in patients with CAD was tested by ROC curves.

### Collection of clinical samples

The clinical study included 50 patients with CAD and 50 control patients treated in Tai’an Central Hospital. The patient’s clinical data are shown in Table 4. All participants underwent coronary angiography, and stenosis was assessed by a cardiologist. Samples meeting the following criteria were included in the CAD group: at least one major coronary artery with stenosis of > 50% [47] and patient age of 50–80 years. The degree of coronary artery stenosis in the control group was < 50%, and the age and sex of the patients in the control group were consistent with those of the patients in the CAD group. Patients with a history of diabetes, cancer, kidney failure, liver disease, or other chronic diseases requiring immunosuppressants, anti-inflammatory drugs, or steroids were excluded. The Medical Ethics Committee of Tai’an Central Hospital approved the research procedure, which was performed in accordance with the Declaration of Helsinki (revised in 2013). All patients provided written informed consent prior to the trial.

**Table 4:** Anthropometric and laboratory profile of study population.

**Parameter**	**Controls (*n* = 50)**	**CAD (*n* = 50)**	***P* value**
Gender (male/female)	23/27	26/24	0.689
Age (years)	63.74 ± 7.95	66.76 ± 7.51	0.073
Hypertension (yes/no)	17/33	26/24	0.106
Smokers (yes/no)	3/47	7/43	0.318
LDL-Cholesterol (mmol/L)	2.47 ± 1.06	2.52 ± 0.87	0.581
HDL-Cholesterol (mmol/L)	1.22 ± 0.38	1.07 ± 0.28	0.103
Total-Cholesterol (mmol/L)	3.91 ± 1.01	4.09 ± 1.08	0.495
Triglyceride (mmol/L)	1.50 ± 0.77	1.70 ± 1.12	0.389
WBC (10^9^/L)	6.11 ± 1.27	6.61 ± 1.71	0.228

### RNA isolation and RT-qPCR

On the second day of admission, 2 mL of fasting venous whole blood was obtained from each patient and placed in ethylenediaminetetraacetic acid anticoagulation tubes. After mixing, 300 μL of whole blood was extracted from each tube. Total RNA was extracted from whole blood cells by TRIpure Reagent LS (Aidlab, Beijing, China). Next, cDNA was synthesized with a HiFiScript gDNA Removal RT MasterMix kit (CoWin Biotech, Jiangsu, China). A MagicSYBR Mixture kit (CoWin Biotech) was used for RT-qPCR. Three duplicate holes were designated for each gene in each sample. GAPDH was used an endogenous control. The specific primers were *HSP90AA1* F: 5′-TATAAGGCAGGCGCGGGGGT-3′, R: 5′-TGCACCAGCCTGCAAAGCTTCC-3′; *CEBPA* F: 5′-GCAAACTCACCGCTCCAATG-3′, R: 5′-TTCTCTCATGGGGGTCTGCT-3′; *FOXO1* F: 5′-AGATGAGTGCCCTGGGCAGC-3′, R: 5′-GATGGACTCCATGTCACAGT-3′; and *GAPDH* F: 5′-CCTCAAGATCATCAGCAATG-3′, R: 5′-CCATCCACAGTCTTCTGGG-3′.

### ELISA and western blot analysis

Three milliliters Ficol-Hypaque (TBD, Tianjin, China) was placed into a 15-ml centrifugation tube. The remaining whole blood samples from the previous step were carefully absorbed and added to the surface of the separation solution. Centrifugation was performed at 550 × *g* on a horizontal rotor centrifuge for 30 min. The first layer obtained by density gradient centrifugation was blood plasma. The expression levels of *HSP90AA1*, *CEBPA*, and *FOXO1* in plasma were detected with an ELISA kit (Meimian, Jiangsu, China). Three duplicate holes were set up for each gene in each sample. The second layer obtained by centrifugation comprised PBMCs. This second layer of cells was gently absorbed, suspended with phosphate-buffered saline, and centrifuged at 250 × *g* for 10 min. If red blood cell precipitation was present, the supernatant was discarded and precipitated with red blood cell lysate (TBD). After the precipitated cells were washed with cold phosphate-buffered saline, 100 μL of cold RIPA lysis buffer (Bestbio, Shanghai, China) containing protease inhibitors was added for oscillatory centrifugation, and the supernatant was obtained. The protein concentration was then determined using a BCA kit (Beyotime, Shanghai, China). The protein (40 μg/lane) was isolated by 7.5% SDS polyacrylamide gel electrophoresis and transferred to an activated polyvinylidene fluoride membrane. After sealing the membrane with 5% skim milk, it was incubated with monoclonal antibodies to CEBPA (1:800, cat# 18311-1-AP; Proteintech, Rosemont, IL, USA), HSP90 (1:2000, cat# 13171-AP; Proteintech), and FOXO1 (1:1000, cat#AB179450; Abcam, Cambridge, UK) at 4°C overnight. GAPDH (1:500, cat#10494-1-AP; Proteintech) was used as the control. Incubation was then performed with horseradish peroxidase-conjugated AffiniPure goat anti-rabbit IgG (H+L) secondary antibody (1:2000, cat#SA00001-2; Proteintech) at room temperature for 1 h. Protein bands were detected using an electrochemiluminescence kit (Proteintech) and chemiluminescence system (Bio-Rad Laboratories, Hercules, CA, USA). All western blot experiments were repeated multiple times.

### Statistical analysis

Statistical analyses were conducted using R version 4.1.2. Student’s *t*-test or the Mann–Whitney *U*-test was used for statistical analysis between two groups based on the normality of data. Data are presented as mean ± standard deviation. The chi-square test was used for categorical variables. *P* < 0.05 was considered statistically significant.
